# When Dryness Extends to the Brain: Brain-Related Non-Sicca Manifestations of Sjögren’s Disease

**DOI:** 10.3390/jcm15103954

**Published:** 2026-05-20

**Authors:** Magdalena Kolanko, Julia Grabowska, Agata Sebastian

**Affiliations:** 1 Wrocław University Hospital, Borowska 213, 50-556 Wrocław, Poland; julia.grabowska27@gmail.com; 2 Department and Clinic of Rheumatology and Internal Medicine, Wroclaw Medical University, Borowska 213, 50-556 Wrocław, Poland; agata.sebastian@umw.edu.pl

**Keywords:** Sjögren’s syndrome, cognitive impairment, brain fog, depression, sleep disturbances, fatigue, sexual dysfunction

## Abstract

**Background:** Sjögren’s disease (SjD) is a chronic systemic autoimmune disorder primarily characterized by lymphocytic infiltration of exocrine glands, leading to xerostomia and xerophthalmia. Beyond glandular involvement, the disease frequently presents with a broad spectrum of systemic and neuropsychiatric manifestations that significantly affect patients’ quality of life. **Methods:** A review of the literature was conducted to identify studies addressing neuropsychological symptoms in patients with SjD. Relevant publications describing cognitive dysfunction, mood disorders, sleep disturbances, fatigue, and sexual dysfunction, as well as potential underlying mechanisms and therapeutic approaches, were included and analyzed. **Results:** Available evidence indicates that neuropsychological symptoms are common among patients with SjD. Cognitive impairment, often described as “brain fog”, may involve deficits in memory, attention, and executive functioning. Depression and anxiety appear to occur more frequently than in the general population and may interact with chronic fatigue and sleep disturbances, contributing to functional impairment. While somatic causes of sexual dysfunctions such as vaginal dryness are well recognized, psychological and psychosexual aspects, including reduced sexual desire, have received comparatively little attention. The pathogenesis of these manifestations is likely multifactorial and may involve immune-mediated processes, cytokine dysregulation, neuroendocrine alterations, microvascular changes, and psychosocial factors. **Conclusions:** Neuropsychological manifestations represent a significant component of the overall disease burden in SjD. Increased awareness and multidisciplinary management strategies may help improve symptom recognition, patient care, and quality of life.

## 1. Introduction

Sjögren’s disease (SjD), previously termed Sjögren syndrome or Sjögren’s syndrome [1], was first described in the late 19th century as a clinical entity manifesting either as a benign sicca syndrome or as a lymphoid malignancy. Reported prevalence estimates vary widely—from 1 to 72 cases per 10,000 individuals—primarily due to methodological heterogeneity across studies, including differences in classification criteria, diagnostic approaches (e.g., symptom-based vs. serologic or biopsy-confirmed cases), and population characteristics. Lower prevalence estimates are typically derived from studies employing stringent classification criteria, while higher estimates often reflect the use of broader, symptom-based case definitions in population-level assessments. The incidence increases with age, reaching its peak in women aged 55–64 years and in men aged 65–74 years [1,2].

The pathogenesis of SjD is multifactorial, with environmental, genetic, neuroendocrine, and immune mechanisms—particularly those involving immune cell activation and cytokine dysregulation—contributing to disease development [2]. Despite extensive research, its underlying mechanisms remain incompletely understood, and reliable clinical diagnosis continues to pose challenges [3]. The 2016 EULAR/ACR classification criteria for SjD rely on serologic detection of anti-SSA (anti-Ro) antibodies, histopathologic assessment of minor salivary gland biopsy, the Schirmer test, unstimulated whole saliva flow measurement, and ocular surface staining [4].

Historically, SjD was categorized as either “primary” (approximately 40% of cases, occurring without an underlying rheumatic condition) or “secondary” (associated with disorders such as rheumatoid arthritis (RA) or systemic lupus erythematosus (SLE)) [5]. In accordance with the 2023 International Rome consensus, these terms are no longer recommended. Instead, the descriptor “associated” should replace “secondary”, a convention adopted throughout the present work [1].

Sjögren’s disease is a systemic autoimmune disorder characterized by a wide spectrum of clinical presentations, varying from isolated exocrine gland dysfunction to extensive multisystem involvement and potentially serious complications [6,7,8]. The neurological manifestations of SjD are collectively referred to as Neuro-Sjögren [9]. The literature indicates that neurological manifestations occur in 8.5–70% of SjD patients [10,11]. The reported frequency of their occurrence varies between studies due to differing definitions of SjD, varying definitions of neurological manifestations, and unequal access to specialized neuroimaging techniques in clinical settings. In at least 25% of cases, neurological symptoms appeared approximately two years before the diagnosis of SjD. In the remaining cases, manifestations of the nervous system developed later, typically 6–7 years after disease onset [10]. However, these estimates should be interpreted with caution, as they are largely derived from a single frequently cited source (Fauchais AL et al., 2012) [12], and their generalizability may be limited by the study design and the heterogeneity of patient populations.

Neurological manifestations in SjD can affect either the central or peripheral nervous system. The peripheral nervous system is more commonly involved [13,14]. Reported rates of CNS involvement vary widely (10–60%) [15], reflecting differences in study design and diagnostic criteria. However, this contrasts with the frequent absence of abnormalities on conventional MRI, highlighting a diagnostic gap and limitations of standard imaging in detecting subtle CNS involvement in SjD.

The most common central nervous system manifestations were headaches, cognitive impairment and depression [9]. Nevertheless, this observation is based on a single study of patients with established Neuro-Sjögren, which limits its generalizability to the broader SjD population. Other potential manifestations of central nervous system involvement may include meningitis, epileptic seizures, transverse myelitis, optic neuritis, ataxia, encephalopathy, and multiple sclerosis-like (MS-like) lesions, as well as various forms of parkinsonism. Psychiatric manifestations are also significant and include mood disorders such as depression and anxiety, as well as psychosis, schizophrenia-like symptoms, dementia, bipolar disorder, and personality disorders [14]. Due to the wide range of symptoms, patients are often managed not only by a rheumatologist but also by a neurologist and psychiatrist. In some cases, these manifestations precede the onset of SjD, which can lead to delayed diagnosis. Taken together, these observations support the concept that in some patients, the impact of SjD may metaphorically “extend to the brain,” contributing to a broad spectrum of non-sicca manifestations. The evidence base for these manifestations is highly heterogeneous, ranging from well-characterized clinical symptoms with consistent reporting (e.g., fatigue and mood disorders) to emerging or poorly defined associations supported by limited or methodologically variable studies (sexual dysfunction, psychiatric associations beyond depression and anxiety).

The aim of this review was to evaluate the current literature on brain-related non-sicca manifestations in patients with SjD, focusing on cognitive impairment, depression, anxiety, sleep disturbances, fatigue, and sexual dysfunction. Specifically, we aimed to assess their prevalence, underlying pathophysiological mechanisms, diagnostic and assessment methods, impact on patients’ quality of life, and therapeutic options, including comparisons with healthy controls and other rheumatic conditions where available.

## 2. Materials and Methods

**Figure 1:**
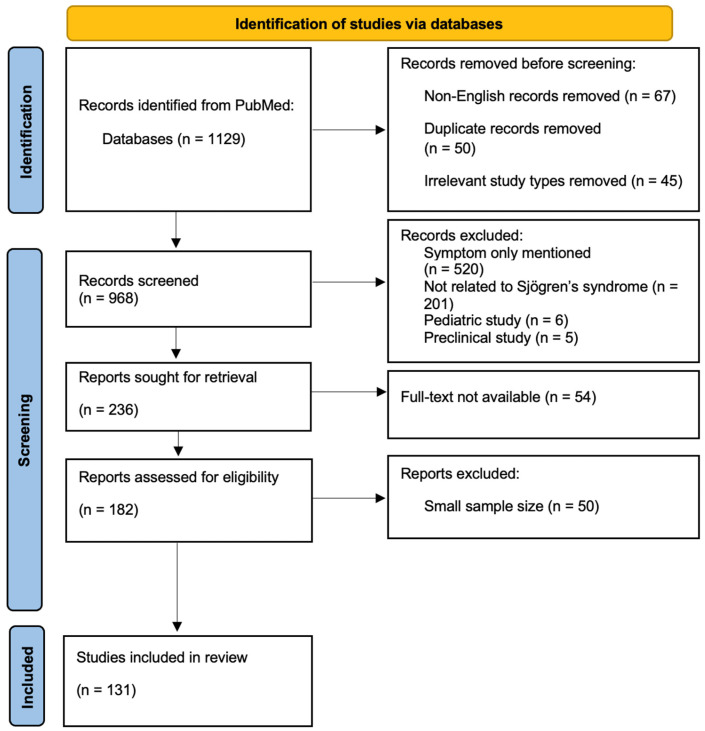
PRISMA flow diagram of the study selection process.

A literature search was performed in the PubMed database between November 2025 and February 2026. The following terms were used: “Sjögren’s syndrome” AND (“cognitive impairment” OR “cognitive dysfunction” OR “brain fog” OR “memory impairment” OR “dementia”), “Sjögren’s syndrome” AND (“depression” OR “anxiety”), “Sjögren’s syndrome” AND (“sleep disturbances” OR “insomnia” OR “sleep apnea” OR “somnolence” OR “restless legs syndrome” OR “narcolepsy”), “Sjögren’s syndrome” AND (“fatigue”), and “Sjögren’s syndrome” AND (“sexual dysfunction” OR “sexual disorders”). The selection process of studies was performed according to PRISMA recommendations and is presented in the PRISMA flow diagram (Figure 1). Searches were limited to articles published in English.

Studies were eligible if they involved adult patients (≥18 years) diagnosed with SjD and addressed at least one of the selected neuropsychological manifestations. Eligible study types included systematic reviews, meta-analyses, clinical trials, observational studies, and clinical guidelines. Case reports, bibliometric reviews, studies involving pediatric populations, studies involving small patient cohorts (fewer than 30 participants), and studies involving animal experiments without clinical relevance were excluded. No formal structured quality appraisal was performed. However, study design, sample size, and methodological limitations were considered qualitatively when interpreting the strength of the available evidence.

Titles and abstracts were initially screened for relevance, followed by full-text evaluation according to predefined inclusion criteria. The study selection process was performed by a single reviewer; therefore, inter-rater agreement statistics were not applicable. To minimize potential selection bias, predefined inclusion and exclusion criteria were strictly applied during title/abstract and full-text screening.

No time restriction was applied during the database search. However, particular attention was given to publications from the last five years (2021–2026) to capture the most recent evidence regarding epidemiology, pathophysiology, assessment, and management of neuropsychological manifestations in SjD. Earlier studies were included only when they met at least one of the following predefined criteria: (i) provided original foundational or first-description data for a given manifestation, (ii) were highly cited landmark studies that established diagnostic or conceptual frameworks, or (iii) addressed clinical or mechanistic aspects not covered by more recent literature. Studies not meeting these criteria were excluded to minimize selection bias.

## 3. Cognitive Impairment in Sjögren’s Disease

### 3.1. Definition, General Background and Prevalence

Cognitive impairment is a broad term referring to disturbances in mental processes that enable learning, thinking, memory, attention, concentration, and decision-making [16,17,18]. The severity of cognitive deficits ranges from subtle changes, commonly classified as mild cognitive impairment (MCI), to severe dementia [19]. Clinical manifestations are heterogeneous and may vary considerably between individuals.

The etiology of MCI is multifactorial and includes systemic diseases, neurological disorders, adverse effects of medications, and psychiatric conditions, resulting in substantial variability in clinical presentation and prognosis. Cognitive impairment may also be influenced by potentially modifiable factors, including sedentary lifestyle, poor nutritional status, social isolation, adverse environmental conditions, and genetic susceptibility. These factors may interact with systemic inflammation and vascular dysfunction, contributing to cognitive decline across diverse disease states [20]. The broad etiological spectrum of cognitive impairment underscores the need for comprehensive differential assessment to determine whether cognitive dysfunction is attributable to SjD or to other potentially reversible or unrelated causes.

Although sleep disturbances, fatigue, depression, anxiety, and chronic pain are common accompanying symptoms in patients with cognitive impairment, these factors alone do not fully account for the observed cognitive deficits [21]. However, it is undeniable that they contribute to the worsening of patients’ cognitive functioning. Therefore, it is crucial to assist patients in effectively managing pain, addressing sleep disturbances, and regulating mood.

Cognitive impairment is commonly observed in individuals with SjD. Although it is most often mild, cases of advanced dementia have also been reported [13].

Across studies, the reported prevalence of cognitive impairment in SjD varies widely, ranging from 22% to nearly 60% [13,22,23,24], reflecting differences in study populations, diagnostic criteria, and assessment methods. For comparison, mild cognitive impairment affects up to 19% of adults over the age of 65 years [25]. In a 2020 study by Seeliger et al., approximately 55% of patients with neuro-Sjögren exhibited cognitive impairment, including 38% with mild symptoms and 17% with severe impairment [9].

### 3.2. Clinical Manifestations

#### 3.2.1. “Brain Fog” as a Distinct Clinical Phenomenon

A frequently reported symptom among patients is “brain fog,” a colloquial term used to describe subjective experiences of cognitive clouding, mental slowing, and reduced clarity of thought [13]. Subjective cognitive complaints (SCC) have been reported in 22–64.6% of cases [22,23]. Patients describe them as temporary memory lapses, mental confusion, and a reduced ability to concentrate, organize tasks, or anticipate future events. These symptoms are not consistently accompanied by measurable deficits on standardized cognitive screening tools such as the MoCA or MMSE in all studies. This suggests a potential dissociation between subjective cognitive experiences and objectively assessed cognitive performance in a subset of patients with SjD [24].

#### 3.2.2. Memory Impairment

Patients with SjD, alongside those with systemic lupus erythematosus, report the highest prevalence of memory impairments among autoimmune rheumatic diseases. Memory impairment in patients with SjD is particularly consequential, as it often prevents them from working or returning to work. This is particularly evident in patients who previously held high-responsibility positions; due to memory difficulties, they are no longer able to perform at their pre-illness level, leading many to feel as though they have experienced a professional failure [18].

Memory impairment adversely affects the treatment process. Forgetting to take medications, report symptoms, attend scheduled appointments, or follow medical recommendations can significantly reduce the effectiveness of therapy.

#### 3.2.3. Progression to Dementia

Cognitive impairment in SjD typically remains stable over time, with progression to dementia occurring only in rare cases. This relatively benign long-term trajectory further supports the notion that cognitive dysfunction in SjD differs fundamentally from classical neurodegenerative processes [26].

### 3.3. Pathophysiology

Neuroimaging studies in SjD patients with cognitive complaints have reported white-matter lesions, altered hippocampal connectivity, and microstructural changes on diffusion tensor imaging. However, after adjustment for confounders such as older age, hypertension, and diabetes mellitus, white-matter abnormalities are often comparable to those observed in control groups, suggesting that a substantial proportion of findings may reflect vascular or age-related comorbidity rather than SjD-specific CNS involvement. In addition, most lesions show no contrast enhancement, supporting a nonspecific microangiopathic or degenerative rather than active inflammatory origin. Although patients with white-matter lesions tend to perform worse on cognitive testing, the clinical significance of these findings remains variable [13]. Therefore, alternative causes, particularly cerebral small-vessel disease, should be considered before attributing MRI abnormalities to SjD.

Resting-state fMRI studies, including data from Hu et al. (2025), suggest altered functional connectivity in frontal, striatal, supplementary motor, and precuneus regions, with some correlations to cognitive performance [15]. However, these findings are preliminary, derived from relatively small cohorts, and require replication.

Consistently, several regions (particularly the frontal cortex, hippocampus, and cerebellum) have been implicated in cognitive dysfunction in SjD [27,28,29], although evidence remains heterogeneous and largely associative. Similarly, neurofilament light chain may reflect CNS involvement [30], but its clinical utility is not yet established.

Overall, conventional MRI is frequently normal or nonspecific, and current neuroimaging findings should be considered exploratory rather than definitive biomarkers of cognitive dysfunction in SjD.

The etiology of cognitive impairment in SjD has not yet been fully elucidated. Current explanations typically center on three hypotheses: microangiopathy resulting in ischemic lesions, an immune-mediated mechanism and intracranial mononuclear infiltration [31].

The first hypothesis proposes that inflammation of small blood vessels leads to their structural damage. The vessel walls thicken and lose elasticity, while the lumen narrows. These changes disrupt blood flow, resulting in local cerebral ischemia, which may in turn cause neurological symptoms [32].

The second hypothesis suggests that cognitive impairment results from the action of anti-Ro and anti-La antibodies, which are characteristic of SjD, as well as anti-neuronal antibodies directed against the patient’s own neural cells. These antibodies may directly damage neurons [26,33].

When the blood–brain barrier is disrupted, infiltration of inflammatory cells occurs. The third hypothesis concerns mononuclear infiltrates, referring to the presence of lymphocytes and monocytes in the central nervous system. These cells induce inflammation, which in turn leads to neurological symptoms [32].

In SjD, neurotransmitter dysregulation may also occur. Elevated levels of proinflammatory cytokines, such as interferon-γ (IFN-γ), interleukin-1 (IL-1), and tumor necrosis factor alpha (TNF-α), stimulate the activity of the enzyme indoleamine 2,3-dioxygenase (IDO), which converts tryptophan into kynurenine and its metabolites. As a result, less tryptophan is available for serotonin production in the central nervous system. Serotonin deficiency in the hippocampus contributes not only to cognitive dysfunction but also to the development of depression [26].

Cognitive impairment reported in patients with SjD should be interpreted within a broader clinical context, as it may arise from multiple, partially overlapping mechanisms. In addition to potential immune-mediated central nervous system involvement, cognitive symptoms may also reflect the cumulative burden of chronic systemic disease, including persistent pain, fatigue, sleep disturbances, and affective disorders such as depression and anxiety. These factors can significantly influence attention, processing speed, and memory, and may mimic or amplify “brain fog”-like complaints. Therefore, distinguishing primary neuroinflammatory mechanisms from secondary, functional, or psychosocial contributors remains challenging in routine clinical practice and is not consistently addressed across studies.

### 3.4. Assessment and Screening Tools

In studies investigating cognitive impairment in patients with SjD, a wide variety of neuropsychological tests have been employed to assess both global cognition and specific cognitive domains. Among these, the most used screening instruments are the Mini-Mental State Examination (MMSE) and the Montreal Cognitive Assessment (MoCA). Studies have shown that the MoCA is more sensitive than the MMSE in detecting subtle cognitive impairment, likely due to its broader and more demanding assessment of executive, visuospatial, and higher-level language functions [34]. In the study conducted by Kooshki et al. in 2025, patients with SjD showed significantly lower MoCA and Serial Digit Learning Test scores compared with healthy controls, whereas no significant difference was observed in MMSE scores between the two groups [35]. Another comprehensive assessment is the CERAD-PLUS, an established cognitive test battery originally developed for Alzheimer’s disease screening, which evaluates executive functions, visuospatial abilities, language, and memory through twelve subtests [9].

There are tests specifically designed to assess individual components that together constitute cognitive function. A key instrument for assessing memory in patients with SjD is the Everyday Memory Questionnaire-Revised (EMQ-R). In the study conducted by Varshney et al. in 2025, EMQ-R scores were positively correlated with overall disease activity, anxiety, depression, fatigue, and pain, while showing a negative correlation with well-being, indicating that greater memory difficulties are associated with lower quality of life [18].

Attention and information processing speed might be assessed using the Digit Symbol Test (DST), while psychomotor abilities might be evaluated with the Number Connection Test–Type A (NCT-A) [15].

### 3.5. Impact on Daily Functioning and Quality of Life

Cognitive impairment has a profound impact on patients’ daily functioning and overall quality of life. In the study conducted by Perella et al. in 2023, cognitive disorders were described by patients as more bothersome than physical fatigue [23]. Difficulties with memory, attention, and executive functioning impair occupational performance, limit independence, and interfere with social relationships.

Despite the common occurrence of cognitive impairment and its significant impact on patients’ quality of life, it is not included in the disease activity scoring system for SjD, namely the EULAR Sjögren’s Syndrome Disease Activity Index (ESSDAI). Notably, the severity of cognitive impairment shows a positive correlation with disease activity as measured by the ESSDAI, whereas it is not associated with disease duration, age, or sex [9,13].

Patients report fluctuating cognitive difficulties with periods of improvement. However, cognitive functioning has never returned to the pre-disease level [18].

Beyond reducing patients’ quality of life, cognitive dysfunction can prevent them from maintaining employment, thereby negatively impacting their economic status [18]. Cognitive impairments also impose a greater burden on healthcare systems, due to increased medical visits, diagnostics, and care requirements [21].

In the study conducted by Varshney et al. in 2025, patients experiencing cognitive impairment reported feeling “stupid,” “embarrassed,” and “judged.” This sense of shame leads many to avoid social situations and gradually withdraw from others, as they feel inferior and out of place. Social isolation negatively affects disease prognosis and significantly reduces overall quality of life. Importantly, despite the substantial impact of cognitive impairment on daily functioning, patients often do not report this symptom to their treating physician. Many believe that, from the clinician’s perspective, such difficulties are less significant than physical manifestations that are more visible and easier to assess [18].

### 3.6. Management and Therapeutic Considerations

At present, no specific therapy is available. Due to the wide range of symptoms and the variability in the severity of cognitive impairment among patients with SjD, treatment should be individualized. Supportive care is recommended, along with continued management of the underlying disease using immunosuppressive agents [13].

## 4. Depression in Sjögren’s Disease

### 4.1. Definition, General Background and Prevalence

Depression is a mood disorder defined by prolonged feelings of sadness and diminished interest or pleasure in previously rewarding activities, which significantly interferes with daily functioning and is linked to reduced quality of life, impaired social and occupational performance, and adverse health outcomes [36,37].

According to the World Health Organization (WHO), an estimated 4% of the population experiences depression, including 5.7% of adults (4.6% among men and 6.9% among women).

Over the past decade, accumulating evidence has highlighted a clear association between autoimmune disorders and depression, with patients suffering from connective tissue diseases exhibiting particularly high rates of depressive symptoms [38]. In the context of SjD, estimates indicate that 32–45.8% of patients experience depression [39,40,41,42,43,44].

In a comparative cross-sectional study by Beider et al., depressive symptoms were most common in patients with SjD (36.3%), compared with systemic lupus erythematosus (28.8%) and rheumatoid arthritis (22%) [45].

While SjD predominantly affects women, there is limited research exploring depression in men presenting with sicca symptoms. A study by Nortey et al. (2022) investigated 98 men with confirmed SjD to examine the relationship between sicca manifestations and depressive symptoms. The results showed that ocular symptoms, such as burning sensations in the eyes, were associated with increased odds of depression in both sexes: men reporting ocular burning had 2.86 times higher odds of being classified as depressed, compared to 2.25 times for women. Interestingly, oral symptoms appeared to be a stronger predictor in men. Among men reporting a “dry mouth,” the odds of depression were 4.88 times higher, whereas women with similar symptoms had 2.13 times higher odds [38]. These findings indicate that oral sicca symptoms may be a particularly salient indicator of depression in men with SjD. Because men are less inclined than women to pursue help for depressive symptoms, clinicians should actively screen male patients and offer targeted interventions to address their mental health needs [46].

### 4.2. Pathophysiology

The pathophysiology of depression in patients with SjD is complex, multifactorial, and not yet fully elucidated. It involves an interplay between well-established vulnerability factors for depression: negative social and environmental factors, such as increased economic burden and lack of social support [47]; the physical burden of SjD, encompassing fatigue, cognitive impairment, sicca manifestations, and autonomic dysfunction [48]; personality traits such as neuroticism, psychoticism, and obsessive tendencies and autoimmune mechanisms [49]. However, interpretation of these mechanisms is complicated by the substantial and often underappreciated confounding effect of chronic disease burden. In many patients, depressive symptomatology is more plausibly interpreted as a secondary, reactive response to persistent pain, functional limitation, and reduced quality of life rather than evidence of a primary neuropsychiatric autoimmune process. Clinically, primary neuropsychiatric SjD is more likely in the presence of objective central nervous system involvement (e.g., focal neurological deficits, neuroimaging or cerebrospinal fluid abnormalities, or vasculitic features), whereas secondary symptoms are typically diffuse and more closely related to pain, fatigue, and psychosocial stressors. However, substantial overlap between these presentations exists, and in routine clinical practice a clear separation is often difficult to achieve. Critically, disentangling primary neuropsychiatric disease from secondary, burden-related affective symptoms remains a major clinical and methodological challenge. This distinction is not consistently defined or applied in the literature, which limits causal inference and contributes to conceptual heterogeneity within the field.

In SjD, an imbalance between proinflammatory cytokines, such as IL-1β, IL-6, IL-12, TNF-α, and IFN, and anti-inflammatory cytokines, including IL-4 and IL-10, plays a key role [50]. In the study by Mrsić et al., it was concluded that elevated IL-6 levels may serve as a predictor of depression [51].

Proposed mechanisms of cytokine influence on the development of depression in SjD patients:

1. Elevated proinflammatory cytokines may also activate the indoleamine 2,3-dioxygenase enzyme, diverting tryptophan toward kynurenine production rather than serotonin synthesis in the central nervous system, potentially contributing to depression and cognitive impairments [52].
2. Proinflammatory cytokines can disrupt the Hypothalamic-Pituitary-Adrenal (HPA) axis, which regulates stress, immunity, and mood. This disruption may contribute to anxiety and depression, while HPA axis dysfunction can further increase cytokine production, creating a feedback loop that worsens immune and neuropsychiatric symptoms [53,54].
3. Increased levels of proinflammatory cytokines can compromise endothelial function, leading to reduced blood flow in white matter and impairing neurons that regulate emotions [55].

Elevated anti-NR2 antibodies in SjD are linked to hippocampal atrophy, contributing to cognitive and mood disturbances [56,57]. Rarely, anti-NMDAR encephalitis may present solely with psychiatric symptoms, which respond to immunotherapy [58]. Xia et al. described a patient with long-term major depression who was later found to have anti-NMDAR encephalitis, with anti-NR1 IgG antibodies in cerebrospinal fluid, indicating a possible link between these antibodies and depressive symptoms [59]. However, anti-NR2 and anti-NMDAR antibodies are rare and not routinely used in clinical practice; they remain primarily research biomarkers, limiting their applicability in routine diagnostics.

Frequent central nervous system white matter lesions have been reported in SjD and may be associated with depression [60,61]. Microstructural alterations and reduced functional connectivity have also been observed in the somatosensory cortex and corticospinal tract [62], while microvascular and ischemic lesions may further impair brain function [63].

Our findings reinforce existing evidence, yet the underlying mechanisms of depression in SjD remain incompletely understood, and further research is needed.

### 4.3. Assessment and Screening Tools

The evaluation of depressive disorders in both research and clinical settings frequently relies on standardized instruments designed to assess symptom severity and identify individuals at risk. However, no depression scale has yet been specifically validated for patients with SjD, and this limitation should be clearly acknowledged, as it constrains the interpretation of all available measures in this population. Commonly used instruments include the Patient Health Questionnaire-9 (PHQ-9) [64], the Hamilton Depression Rating Scale (HAMD-17) [65], and the Beck Depression Inventory (BDI) [66], each differing in administration, length, and psychometric properties.

Results showed that the PHQ-9 was satisfactory in terms of reliability, validity and distinguishing the severity of depression. It is a simple, rapid, effective and reliable tool which can be used as an alternative to the HAMD-17 to assess the severity of depression [65]. Pending the results of further studies, the attributes of the PHQ-9, of being shorter and based on the diagnostic criteria for depression, may indicate an advantage over the BDI-II [66]. Other instruments, including the Centre for Epidemiological Studies Depression Scale (CES-D), Quick Inventory of Depressive Symptomatology (QIDS), Geriatric Depression Scale (GDS-15), and Montgomery-Åsberg Depression Rating Scale (MADRS), are often applied in research settings depending on study population and objectives [67,68].

Notably, many of these scales include items assessing fatigue, sleep disturbances, and cognitive symptoms—features that overlap with core manifestations of SjD—which may contribute to an overestimation of depressive symptom burden in this population. Although the magnitude of this potential bias has not been consistently quantified, available evidence from studies in chronic somatic conditions suggests that somatic item overlap can meaningfully influence total scores and inflate prevalence estimates. To mitigate this limitation, some authors have proposed the use of adjusted scoring approaches (e.g., excluding or down-weighting somatic items), complementary assessment strategies focusing on affective and cognitive symptoms, or the combination of self-report scales with clinical evaluation; however, these approaches have not yet been systematically validated in patients with SjD.

### 4.4. Impact on Daily Functioning and Quality of Life

Rheumatic diseases are frequently associated with early retirement and increased reliance on social benefits. In a large cross-sectional study including 445 patients with rheumatoid arthritis (36.9%), systemic lupus erythematosus (32.8%), and Sjögren’s disease (30.3%), Beider et al. reported that rates of premature retirement were particularly high in SLE and SjD. Specifically, 85% of patients with SLE (41/48) and 66% of patients with SjD (40/61) reported early retirement due to their illness. In comparison, 49% of patients with RA (37/76) experienced early retirement, representing a statistically significant difference (*p* = 0.001). In addition, individuals who retired prematurely because of their disease were more likely to experience depression. While the study provides evidence of a strong association between disease burden and work disability, these findings should be interpreted in the context of the study population and its cross-sectional design. Notably, within the SjD subgroup, all patients who retired early (40/40) indicated their underlying disease as the direct cause of retirement [45].

In patients with SjD, depression is a major contributor to fatigue and frequently exacerbates sleep disturbances, leading to a significant decline in overall quality of life even with patients with SjD but without depression [47,69].

Despite advances in medical management, addressing the psychosocial needs of patients with SjD is essential for improving overall quality of life and patient well-being.

### 4.5. Management and Therapeutic Considerations

Pharmacological treatments, including anti-inflammatory drugs, have shown mixed results due to variations in study design, patient baseline inflammation, and dosing regimens, highlighting the need for standardized clinical trials [70,71]. To specifically address depression and mental fatigue, supplementing treatment with antianxiety or antidepressant medications, such as paroxetine, is recommended [48]. Traditional Chinese medicine has also demonstrated potential benefits for mood regulation, offering additional avenues for clinical practice [71]. Effective management of depression in SjD requires a multifaceted approach. Psychological interventions, patient education, and exercise therapy can improve mood, reduce fatigue and pain, and enhance overall quality of life.

## 5. Anxiety in Sjögren’s Disease

### 5.1. Definition, General Background and Prevalence

Anxiety is a frequent neuropsychiatric comorbidity in SjD. It should not be considered merely a secondary manifestation of depression, as it may represent an independent clinical phenotype with distinct biological and functional correlates.

Reported prevalence rates of anxiety in SjD vary across studies, reflecting differences in assessment tools, population characteristics, and disease severity. Anxiety symptoms have been documented in approximately 18% to 56,6% of patients [21,45,51,72,73]. These findings underscore that anxiety is not an incidental feature but a common and clinically relevant aspect of SjD.

Anxiety in SjD has been associated with several sociodemographic and clinical factors. Lower educational attainment, longer disease duration, and greater systemic disease activity have been more frequently observed among patients with anxiety compared with those without [72]. Importantly, individual psychological vulnerability also appears to modulate the risk of anxiety development. Certain personality traits, particularly high levels of neuroticism, are associated with heightened emotional reactivity, increased sensitivity to internal bodily sensations, and reduced tolerance to stress [49,74]. This predisposition may partly explain the considerable interindividual variability in anxiety severity observed among patients with comparable clinical disease burden.

### 5.2. Clinical Manifestations

Patients with anxiety commonly experience excessive fear, persistent worry, and heightened inner tension. Individuals may also display somatic symptoms such as palpitations, muscle tension, or difficulties with concentration. In SjD, anxiety may additionally present alongside obsessive or compulsive features, including intrusive thoughts and repetitive behaviors aimed at reducing distress [75].

### 5.3. Pathophysiology

The pathogenesis of anxiety in SjD is likely multifactorial, involving immune, neuroendocrine, and psychosocial mechanisms. Notably, anxiety symptoms have been reported more frequently in patients with the presence of anti-NR2 antibodies. Anti-NR2 antibodies have been implicated in hippocampal atrophy, a brain structure critically involved in the modulation of stress responses and emotional regulation [74]. This immune-mediated neurobiological mechanism provides a plausible link between autoantibody activity and anxiety manifestations in SjD. Growing evidence suggests that alterations in gut microbiota may play a contributory role [72,75]; however, the relationship between gut microbiota and anxiety is still considered preliminary, largely based on associative and experimental data, and should be interpreted as hypothesis-generating rather than conclusive.

### 5.4. Assessment and Screening Tools

In studies investigating anxiety in patients with SjD, several standardized questionnaires have been employed to assess anxiety symptoms. These include the State-Trait Anxiety Inventory (STAI), which allows differentiation between situational anxiety and more stable anxiety traits [24,76], as well as other commonly used instruments such as the Self-Rating Anxiety Scale (SAS) [15], the Generalized Anxiety Disorder 7-item scale (GAD-7) [73], and the Hospital Anxiety and Depression Scale (HADS) [72]. However, these instruments may be affected by symptom overlap inflation, as somatic manifestations of SjD (e.g., fatigue, sleep disturbances, and autonomic symptoms) can contribute to elevated anxiety scores independent of primary anxiety pathology. Based on the available evidence, routine screening for anxiety using validated instruments should be incorporated into the standard clinical assessment of patients with SjD.

### 5.5. Impact on Daily Functioning and Quality of Life

Anxiety has a profound negative impact on quality of life in patients with SjD. Psychological distress may amplify physical symptoms, reduce coping capacity, and impair social and occupational functioning, thereby increasing overall disease burden [24]. Importantly, anxiety may not only arise as a consequence of SjD but may also actively influence disease perception, symptom reporting, and healthcare utilization [39]. Heightened anxiety can increase vigilance toward bodily sensations, leading to an overestimation of disease activity and contributing to a self-perpetuating cycle of psychological distress, physical symptom burden, work disability, and reduced participation in daily activities [49].

### 5.6. Management and Therapeutic Considerations

Management of anxiety in SjD should be comprehensive and multidisciplinary. Early identification and treatment of anxiety symptoms are crucial to improving overall outcomes. Psychological interventions, including individual or group-based psychosomatic therapies, may enhance disease acceptance and coping strategies [45]. A study conducted by de Orleans Casagrande et al. in 2023 also demonstrated that yoga may have a beneficial effect on alleviating anxiety symptoms [77]. Such approaches should be more routinely recommended by treating physicians. Pharmacological treatment may be considered, particularly in cases of moderate to severe anxiety, always with attention to comorbid depression and potential interactions with immunomodulatory therapies [74].

### 5.7. Research Gaps and Future Directions

Despite growing evidence of a high prevalence of anxiety in SjD, longitudinal and interventional studies addressing its pathophysiology and clinical consequences remain scarce. A further limitation in the current literature is that anxiety in SjD is frequently assessed in conjunction with depressive symptoms, with many studies using combined anxiety-depression scales or reporting both constructs together. This limits the ability to disentangle anxiety as an independent clinical phenotype and may contribute to the relative scarcity of anxiety-specific analyses compared with depression-focused research. Future studies should more systematically investigate anxiety as a distinct clinical construct in SjD, rather than as a component of broader psychological distress or combined anxiety-depression measures. This would allow a better characterization of its prevalence, specific correlates, and underlying mechanisms.

## 6. Sleep Disturbances in Sjögren’s Disease

### 6.1. Definition, General Background and Prevalence

Sleep disorders are common worldwide, affecting approximately 30–45% of the adult population at some point of life [78]. Sleep impairment is particularly prevalent in patients with connective tissue diseases (CTD), as more than 60% report poor sleep quality, with no significant differences among CTD subgroups [79].

In SjD, sleep impairment may result from both disease-related symptoms and comorbid conditions such as depression. Nocturnal sicca symptoms, pain, and nocturia frequently disrupt sleep continuity [80,81]. Central sensitization, which contributes to widespread pain in SjD, has been shown to negatively affect sleep quality and should be considered a key underlying mechanism [82]. The prevalence of sleep disorders in SjD ranges from 25% to 82.8% [83,84] and includes insomnia, restless legs syndrome, frequent nocturnal awakenings, obstructive sleep apnea, excessive daytime sleepiness, and periodic limb movements during sleep [85].

Compared with healthy controls and patients with RA, individuals with SjD exhibit a greater sleep deficit, increased nocturnal pain, and more pronounced difficulty initiating and maintaining sleep. Polysomnographic studies demonstrate reduced sleep efficiency, increased wake after sleep onset, and sleep fragmentation, which may contribute to fatigue [81]. Patients with poor sleep quality report higher levels of fatigue [86] and significantly reduced health-related quality of life [87].

Overall, SjD is associated with a broad spectrum of sleep disturbances that interact with pain, mood, and fatigue. Identification and targeted treatment of sleep problems, including behavioral interventions and symptom-directed therapies, may improve physical and cognitive functioning and overall quality of life in patients with SjD.

Despite growing interest in neuropsychiatric and sleep-related manifestations in SjD, several key gaps remain. The relationships between sleep disturbances, fatigue, and depression are often described but not systematically disentangled, limiting causal interpretation. Autonomic dysfunction, although potentially relevant to sleep impairment, is inconsistently assessed and not routinely integrated into clinical studies.

In addition, the distinction between primary neuropsychiatric SjD and secondary, burden-related symptoms is rarely operationalised, despite its clinical importance. Finally, current evidence is largely indirect, and there is a lack of robust, multidimensional studies combining sleep, autonomic, and neuroimaging assessments.

### 6.2. Clinical Manifestations

#### 6.2.1. Insomnia

Insomnia is highly prevalent among patients with SjD, affecting up to 77% of individuals in some cohorts [21]. The prevalence of insomnia in the general population is estimated to be approximately 12.4% [88]. Notably, fatigue—rather than daytime sleepiness—has been identified as a predominant feature of psychophysiological insomnia [89]. Cognitive-behavioral therapy for insomnia (CBT-I) is a first-line treatment for insomnia and has shown efficacy in various chronic conditions [90,91]. However, this evidence is based on general populations, with no SjD-specific interventional trials available. Its use in SjD is therefore extrapolative, although it may be a promising approach to improving sleep quality and reducing fatigue in this group.

#### 6.2.2. Daytime Somnolence

Patients with SjD frequently experience increased daytime sleepiness compared with healthy controls. Excessive daytime sleepiness affects 40–55% of patients and may be related to sleep disorders or poor sleep quality [82,92]. Gudbjörnsson et al. reported that SjD patients were sleepy five times more often than rheumatoid arthritis controls and nearly three times more often than healthy individuals [80].

In the UK Primary Sjögren’s Syndrome Registry, daytime sleepiness was common, with patients reporting notable daytime fatigue. Nocturnal sleep was frequently interrupted, as patients woke up on average twice per night. After initially falling asleep, they spent nearly an hour awake during the night, and overall sleep efficiency was reduced, with patients sleeping effectively for only about two-thirds of the time spent in bed. These sleep disruptions were closely linked to fatigue, and patients continued to experience high levels of orthostatic symptoms, highlighting the substantial impact of sleep disturbances in SjD [93].

These findings indicate that poor sleep and daytime sleepiness are highly prevalent in SjD and closely linked to fatigue and autonomic dysfunction, emphasizing the need for targeted assessment and management of sleep disturbances in this population.

#### 6.2.3. Obstructive Sleep Apnea

Obstructive sleep apnea (OSA) is a sleep-related breathing disorder characterized by recurrent upper airway obstruction and sleep fragmentation, and it is a well-established cause of excessive daytime sleepiness (EDS) [89] and mood variability [85]. However, data on the prevalence and clinical impact of OSA in patients with SjD remain limited.

Although polysomnography-based data suggest a high prevalence of OSA in Sjögren’s disease (SjD), such findings should be interpreted with caution. In the study by Karabul et al., OSA was reported in up to 84% of patients; however, this result is likely influenced by the small sample size and significant selection bias, as participants were recruited from a sleep clinic population rather than a representative SjD cohort [94]. In another study, the prevalence of OSA was approximately twice as high in the SjD group compared to controls. Furthermore, more SjD patients met criteria for obstructive sleep apnea syndrome (OSAS; AHI > 15/h and Epworth Sleepiness Scale [ESS] > 10) than controls. Respiratory and spontaneous arousal indices were elevated in SjD patients, whereas the periodic limb movement arousal index was lower compared with controls. No significant differences were observed in total sleep time or sleep efficiency, although sleep onset latency was prolonged in SjD patients [89].

Another investigation using polysomnography detected OSA in 37 of 44 SD patients (84%), with 25 patients (68%) classified as having moderate to severe OSA. Age, body mass index (BMI), and overweight or obesity were significantly associated with the presence of OSA in these patients [94].

Treatment of OSA with continuous positive airway pressure (CPAP) has been shown to improve self-reported daytime sleepiness and fatigue in the general population, although effects on mood are variable. Preliminary evidence suggests that SjD patients with high frequencies of obstructive apneas and hypopneas may experience substantial improvements in both daytime sleepiness and fatigue when treated with CPAP [85,89].

The role of other risk factors, such as heavy smoking, in OSA among SjD patients has not been adequately studied [95].

Dry mouth, reflecting upper airway dryness, has been proposed as a potential predictor of OSA, although studies remain limited; Jülich et al.’s study included only 14 patients [93]. In the study by Karabul et al., which investigated the frequency of obstructive sleep apnea in patients with SjD, no significant association was observed between dry mouth or ocular dryness and OSA [94]. Larger, well-powered studies are warranted to further explore these relationships.

Finally, OSA is a known contributor to excessive daytime sleepiness in the general population and would be expected to contribute similarly in SjD. However, existing data are inconsistent. For example, the study by Karabul et al. reported no significant correlation between OSA and EDS in SjD patients, despite the presence of EDS in this population [94]. This highlights the need for further research to elucidate the relationship between sleep-disordered breathing and daytime sleepiness in patients with SjD.

#### 6.2.4. Restless Legs Syndrome

Restless Legs Syndrome (RLS) is diagnosed based on the following criteria:

1. An urge to move the legs, usually accompanied or triggered by uncomfortable or unpleasant sensations in the legs.
2. Symptoms begin or worsen during periods of rest or inactivity.
3. Symptoms are partially or completely relieved by movement, at least temporarily.
4. Symptoms are more pronounced in the evening or at night, or occur exclusively during these periods. In severe cases, nocturnal worsening may not be evident but must have been previously present.

In a large survey of primary care patients with frequent RLS symptoms, most reported daytime fatigue, difficulty relaxing, and interference with daily activities. Many also experienced depressive symptoms and impaired concentration the following day, highlighting the substantial functional and psychological burden of RLS [96].

RLS affects approximately 5–10% of the general population and has a profound impact on sleep and daytime functioning [97,98]. RLS is frequently observed in patients with rheumatologic disorders, including SjD, highlighting the importance of awareness among rheumatologists. Given the overlap between RLS symptoms and manifestations of rheumatologic diseases, rheumatologists must be able to recognize, differentiate, and manage RLS effectively [96].

In Aksoy et al., RLS affected 26.7% of SjD patients and was more severe than in controls, with depression and insomnia as independent predictors. The study was a case–control design including 55 patients aged 18–65 diagnosed with SjD according to the 2016 ACR/EULAR criteria and 60 age-matched healthy controls [99].

#### 6.2.5. Autonomic Symptoms

Autonomic nervous system (ANS) activity and sleep are tightly interconnected through shared central regulatory networks, particularly within the hypothalamus and brainstem. Sleep stages are associated with characteristic autonomic patterns, with increased parasympathetic dominance during non-REM sleep and relative sympathetic activation during REM sleep, reflecting a dynamic, state-dependent modulation of cardiovascular and autonomic function. Conversely, autonomic imbalance may disrupt sleep regulation by promoting sleep fragmentation and impairing sleep stability, highlighting a bidirectional relationship between autonomic control and sleep architecture [100,101,102].

In SjD, this relationship may be further influenced by small fiber neuropathy (SFN), which can involve both somatic and autonomic fibers. SFN is associated with impaired autonomic regulation, including sudomotor, cardiovascular, and gastrointestinal dysfunction, and may therefore represent a key peripheral mechanism linking immune-mediated neuropathy with sleep disturbances such as nocturnal arousals and non-restorative sleep [103,104,105].

Nocturnal autonomic symptoms in patients with SjD have been infrequently studied. In the study by Gudbjörnsson et al., which included 40 patients with SjD, 20% of participants reported experiencing nocturnal sweating, compared with 12% in the rheumatoid arthritis comparison group, although no statistically significant difference was observed. Additionally, 5% of SjD patients reported nocturnal palpitations, a symptom not observed in either the RA or healthy control groups [81]. Despite its clinical relevance, it receives considerably less attention than other sleep-related conditions such as restless legs syndrome or narcolepsy. Expansion of this topic is strongly recommended.

#### 6.2.6. Narcolepsy

Narcolepsy is rarely reported in patients with Sjögren’s disease (SjD). In the cohort study by Theander et al., 2 of 72 patients with SjD self-reported a diagnosis of narcolepsy, whereas no cases were reported among healthy controls; however, this outcome was not systematically assessed across studies, limiting firm conclusions [106].

Evidence for a potential association between narcolepsy and SjD is currently limited and largely indirect. While registry-based studies suggest that narcolepsy co-occurs more frequently with autoimmune diseases, including SjD, supporting the hypothesis of shared immunogenetic susceptibility (e.g., HLA-associated risk), these data do not establish disease-specific causality [107].

Several case reports have described narcolepsy in patients with SjD, suggesting a possible but rare association; however, these observations should be interpreted with caution, as case-level evidence was not included in the predefined evidence base of this review.

Although narcolepsy appears to be extremely rare in SjD, it may be considered in patients presenting with excessive daytime sleepiness, particularly when neurological symptoms are present. Systematic evaluation of sleep disorders in this population may help identify atypical manifestations and better define their clinical relevance.

## 7. Fatigue in Sjögren’s Disease

### 7.1. Definition, General Background and Prevalence

Fatigue is commonly defined as a persistent sense of low energy, tiredness, physical weakness, and impaired concentration. Although short-term fatigue lasting less than six months affects approximately 20.4% of the general population, its prevalence is substantially higher among individuals with autoimmune diseases, reaching 60–70% [17]. In SjD, fatigue—alongside pain and sicca symptoms—is among the complaints patients most frequently wish to see alleviated [108].

The pathogenesis of fatigue in SjD is complex and multifactorial. Ayar et al. reported that more than half of patients experienced significant fatigue, with depression, fibromyalgia, disease activity, and insomnia emerging as independent predictors. Among these, depression was identified as the strongest determinant, indicating that depressive symptoms alone may substantially contribute to fatigue burden and represent a key therapeutic target [109]. Hombre-Díaz et al. demonstrated that fatigue and pain were the principal drivers of impaired quality of life in SjD, irrespective of disease activity, demographic characteristics, work disability, or coexisting fibromyalgia [110].

The profound impact of fatigue on functioning and well-being in SjD highlights its importance as a key symptom deserving focused investigation.

### 7.2. Pathophysiology

The biological basis of fatigue in SjD remains elusive and appears to differ from classical inflammatory mechanisms [111]. Accumulating evidence suggests that fatigue-related signaling is more closely linked to cellular protection and defense responses than to pro-inflammatory activity. This concept aligns with the notion of sickness behavior, in which fatigue serves an adaptive role during acute illness, but becomes maladaptive when chronically activated in autoimmune diseases [106,112].

Notably, fatigue in SjD shows little association with systemic disease activity and demonstrates a paradoxical inverse relationship with pro-inflammatory cytokines [111,113]. Several studies have shown that although inflammatory cytokines such as TNF-α, IFN-γ, IP-10, and LT-α are elevated in SjD compared to controls, higher cytokine levels are associated with lower patient-reported fatigue [114,115]. These findings challenge the traditional inflammation-driven model of fatigue and suggest that individual susceptibility—potentially shaped by genetic or epigenetic factors—may determine fatigue persistence, even in the absence of active inflammation.

### 7.3. Assessment and Screening Tools

Two instruments specifically designed for SjD are commonly used to assess fatigue. The Profile of Fatigue and Discomfort-Sicca Symptoms Inventory (PROFAD-SSI) includes the fatigue component (Profile of Fatigue-ProF) that evaluates both somatic (ProF-S) and mental (ProF-M) fatigue [116]. The EULAR Sjögren’s Syndrome Patient Reported Index (ESSPRI) employs 0–10 scales to capture patient-reported fatigue, dryness, and musculoskeletal pain [117].

For a simpler approach, the 10 cm visual analogue scale (VAS) provides a single global fatigue score, with higher values indicating greater severity. While VAS is widely used in SjD research due to its ease of use, multidimensional tools offer a more comprehensive assessment, capturing the full spectrum of fatigue and its clinical correlations. In addition, non-disease-specific multi-item questionnaires are often applied when comparing fatigue across different conditions [118].

### 7.4. Management and Therapeutic Considerations

Fatigue in SjD is a complex, multifactorial symptom that is often poorly responsive to immunosuppressive and biological therapy [119,120,121]. A systematic review of randomized trials found no evidence that rituximab is superior to placebo in improving fatigue-related outcomes in SjD. Although some within-group improvements were observed, these were not sustained in placebo-controlled analyses, indicating a lack of robust therapeutic efficacy for this indication [122]. Similarly, in a multicenter randomized placebo-controlled trial conducted by Bowman et al. (2017), rituximab failed to demonstrate clinically meaningful improvements in fatigue in patients with primary Sjögren’s syndrome [120]. A retrospective single-center study suggested that methotrexate may be associated with greater improvement in patient-reported fatigue measures compared with hydroxychloroquine in SjD. However, due to the non-randomized design and limited methodological rigor, these findings should be interpreted as preliminary and do not allow for firm conclusions regarding comparative efficacy [119]. Therapeutic agents such as leflunomide, zidovudine, bortezomib, and total glucosides of paeony, belimumab, epratuzumab, abatacept, etanercept, and anakinra remain insufficiently studied in the context of fatigue and other systemic manifestations of SjD, and therefore their clinical utility requires further rigorous evaluation. In contrast, other proposed interventions, including dehydroepiandrosterone (DHEA), gamma-linolenic acid, doxycycline, and infliximab, have not demonstrated consistent or clinically meaningful efficacy across available studies, and are not supported by current evidence for routine use in this indication [123]. 

Current management primarily focuses on symptom relief and improving quality of life rather than targeting a single biological pathway. Non-pharmacological interventions, such as aerobic exercise, cognitive-behavioral therapy, sleep hygiene, and energy conservation strategies, have shown benefit in reducing fatigue and enhancing daily functioning [111,123]. A holistic, multidisciplinary approach is therefore recommended, combining lifestyle modifications, psychological support, and careful management of comorbidities such as pain, depression, and sleep disturbances.

## 8. Sexual Disorders in Sjögren’s Disease

### 8.1. Definition, General Background and Prevalence

Sexual disorders in SjD are commonly attributed to peripheral sicca manifestations, particularly vaginal dryness leading to dyspareunia. The fear of pain alone can discourage patients from engaging in sexual activity [121]. Increasing evidence indicates that sexual dysfunction in this population extends beyond local symptoms and involves significant neuropsychological and emotional components [124].

Studies have shown that patients with SjD are generally less sexually active compared with control groups [125]. Patients frequently report reduced sexual desire, impaired arousal, and diminished sexual satisfaction. While these symptoms are consistent with features of hypoactive sexual desire disorder (HSDD) described in the general population, direct evidence linking SjD-specific mechanisms to formal HSDD diagnosis remains limited [124]. Evidence from general HSDD literature, including reviews and meta-analyses conducted in non-SjD populations, suggests that hormonal imbalances, such as relatively low androgen levels, may play a role in sexual dysfunction [126]; however, these findings should be interpreted cautiously when extrapolated to patients with SjD due to the lack of disease-specific validation. 

Oral dryness may further interfere with intimacy, whereas disease-related changes in body image negatively affect sexual self-perception [127]. Sexual difficulties tend to evoke stronger emotional responses than other symptoms, highlighting their profound psychological impact [121]. The psychological sphere is additionally influenced by chronic pain, fatigue, depression and anxiety, which are commonly present in patients with SjD [124]. In the study conducted by Gözüküçük M. et al. (2023), depression and anxiety scores were significantly higher among women with sexual dysfunction in the SjD group. However, no relationship was found between disease activity, medications used and sexual dysfunction [128].

In the general population, the prevalence of HSDD is estimated at approximately 12% based on meta-analytic data [129]. In contrast, studies focusing on women with SjD report markedly higher rates of sexual disorders, ranging from 78.5% to 85.2% [121,130]. The prevalence also varies with age, with younger women reporting fewer problems and older women experiencing more, a pattern that mirrors observations in the general population [128].

Data on sexual dysfunction in men with SjD are extremely limited, primarily due to the low number of affected male patients. In the study conducted by Rebel D et al. (2025) on a sample of nine men, 67% were found to have sexual disorders [127]. These findings highlight the need to expand research on sexual health in men with SjD.

### 8.2. Assessment and Screening Tools

Despite its high prevalence and substantial psychosocial impact, sexual dysfunction remains underreported by patients and insufficiently addressed in clinical practice [125]. Proactive assessment using validated instruments is therefore warranted. A useful assessment tool is the Female Sexual Function Index (FSFI), which evaluates multiple domains of sexual function, including desire, arousal, orgasm, lubrication, satisfaction, and pain [124]. Another useful questionnaire is the Female Sexual Distress Scale (FSDS), which assesses the personal distress associated with sexual dysfunction [125]. Men can be assessed using the International Index of Erectile Function (IIEF) questionnaire [127].

### 8.3. Management and Therapeutic Considerations

Management of sexual dysfunction in SjD is challenging due to its multifactorial nature. Although sicca-related symptoms may be partially alleviated with lubricants, psychological and neuropsychiatric aspects are more difficult to address and remain underrepresented in therapeutic strategies. Multidisciplinary approaches involving gynecological care and psychotherapeutic support may be beneficial. Interventions such as mindfulness techniques can also provide beneficial effects. The quality of the relationship with a partner plays a significant role, making couples therapy a potentially effective intervention to improve the situation [124]. The study conducted by Kurt et al. (2023) demonstrated that pelvic floor training reduces sexual disorders and may be incorporated into the rehabilitation of patients with SjD [131].

### 8.4. Research Gaps and Future Directions

A fulfilling sexual life depends on both physical and psychological factors, both of which may be disrupted in patients with SjD. Overall, current research on sexual dysfunction in SjD remains limited, with a predominant focus on sicca symptoms and small study populations. Greater attention to neuropsychological mechanisms and inclusion of both sexes in future studies are essential to improve understanding and management of this clinically relevant yet frequently overlooked aspect of the disease.

## 9. Limitations

Several limitations should be acknowledged when interpreting the findings summarized in this review. 

First, the literature search was conducted using a single database, which may have led to the omission of relevant studies indexed elsewhere and introduced a risk of selection bias. In addition, the screening and selection of studies were performed by a single reviewer, which may further increase the potential for selection bias despite the use of predefined inclusion and exclusion criteria.

Second, the available literature on neuropsychiatric manifestations of SjD is highly heterogeneous with regard to study design, patient populations, and diagnostic criteria. Many studies involve relatively small sample sizes and cross-sectional designs, which limits the ability to establish causal relationships between disease mechanisms and neuropsychiatric symptoms.

Third, no formal structured quality appraisal was performed. As a result, the strength of the included evidence could not be systematically graded, and study quality was assessed only qualitatively based on design and methodological considerations. Finally, different studies use a wide range of screening tools and questionnaires to assess cognitive impairment, mood disorders, sleep disturbances, and fatigue. This methodological variability may contribute to the broad range of reported prevalence rates and complicates direct comparisons across studies. 

Several areas remain insufficiently investigated, particularly sexual dysfunction and neuropsychiatric manifestations in men with SjD. Importantly, this reflects a broader structural gap in the literature, as most available evidence on sexual dysfunction in SjD is derived from female cohorts, limiting the generalisability of current findings to male patients. Further large-scale longitudinal studies using standardized assessment methods are needed to better understand the mechanisms and clinical impact of these manifestations.

## 10. Conclusions

SjD is a complex systemic autoimmune disorder whose clinical impact extends far beyond the classical sicca symptoms. Neuropsychological manifestations—including cognitive impairment, depression, anxiety, sleep disturbances, fatigue, and sexual dysfunction—are highly prevalent and substantially contribute to the overall disease burden and reduced quality of life experienced by patients. Importantly, these symptoms often do not respond adequately to standard therapies targeting glandular involvement.

The pathogenesis of these symptoms is multifactorial and likely involves an interplay of immune dysregulation, cytokine imbalance, neuroendocrine disturbances, microvascular changes, and psychosocial factors. Importantly, many of these manifestations remain underrecognized and underreported in clinical practice, partly because they are not routinely included in disease activity assessments and may overlap with other symptoms of the disease. Taken together, these observations support the concept that SjD is not confined to exocrine gland dysfunction, but may extend beyond classical “sicca” manifestations to involve the central nervous system. It is consistent with the idea that in SjD, “dryness extends to the brain,” as reflected in its neuropsychological burden.

Given their significant impact on daily functioning, mental health, and social well-being, greater clinical awareness and systematic screening for neuropsychological symptoms should be incorporated into the routine care of patients with SjD. A multidisciplinary approach involving rheumatologists, neurologists, psychiatrists, psychologists, and other specialists may improve symptom recognition and management. 

Future research should focus on large, well-designed longitudinal studies to better clarify the underlying mechanisms of neuropsychological manifestations in SjD and to develop targeted therapeutic strategies aimed at improving both clinical outcomes and patients’ quality of life.

## Data Availability

No new data were created or analyzed in this study. Data sharing is not applicable to this article.
